# Comparison of FDG-PET/CT and CT for evaluation of tumor response to nivolumab plus ipilimumab combination therapy and prognosis prediction in patients with unresectable malignant pleural mesothelioma

**DOI:** 10.18632/oncotarget.28594

**Published:** 2024-06-20

**Authors:** Kazuhiro Kitajima, Kozo Kuribayashi, Toshiyuki Minami, Hiroyuki Yokoyama, Akifumi Nakamura, Masaki Hashimoto, Takashi Kijima, Seiki Hasegawa, Hayato Kaida, Koichiro Yamakado

**Affiliations:** ^1^Department of Radiology, Hyogo Medical University, Nishinomiya, Hyogo, Japan; ^2^Division of Respiratory Medicine, Department of Internal Medicine, Hyogo Medical University, Nishinomiya, Hyogo, Japan; ^3^Department of Thoracic Surgery, Hyogo Medical University, Nishinomiya, Hyogo, Japan; ^4^Department of Radiology, Kindai University Faculty of Medicine, Osaka, Japan

**Keywords:** mesothelioma, immunotherapy, FDG (fluorodeoxyglucose), PET-CT (positron emission tomography-computed tomography), immunotherapy-modified PERCIST (positron emission tomography response criteria in solid tumors)

## Abstract

Objectives: Results for malignant pleural mesothelioma (MPM) patients following first-line treatment with nivolumab plus ipilimumab obtained with immunotherapy-modified PERCIST (imPERCIST), shown by [^18^F]fluorodeoxyglucose positron emission tomography/computed tomography (FDG-PET/CT), and modified RECIST (mRECIST), shown by CT, were compared for response evaluation and prognosis prediction.

Results: imPERCIST indicated nine progressive metabolic disease (PMD), eight stable metabolic disease (SMD), four partial metabolic response (PMR), and five complete metabolic response (CMR) cases. mRECIST showed nine with progressive disease (PD), nine stable disease (SD), seven partial response (PR), and one complete response (CR). Although high concordance was noted (κ = 0.827), imPERCIST correctly judged a greater percentage with CMR (15.4%). Following a median 10.0 months, 15 patients showed progression and eight died from MPM. With both, progression-free survival (PFS) and overall survival (OS) were significantly longer in patients without progression (CMR/PMR/SMD, CR/PR/SD, respectively) as compared to PMD/PD patients (imPERCIST *p* < 0.0001 and *p* = 0.015, respectively; mRECIST *p* < 0.0001 and *p* = 0.015, respectively).

Methods: Twenty-six patients (23 males, 3 females; median 73.5 years) with histologically proven MPM and no curative surgery received nivolumab plus ipilimumab combination therapy. FDG-PET/CT and diagnostic CT scanning at the baseline, and after 2–4 cycles (2 in three, 3 in 17, 4 in six patients) were performed. Therapeutic response findings evaluated using imPERCIST and mRECIST were compared. PFS and OS analyses were done using log-rank and Cox methods.

Conclusion: For unresectable MPM patient examinations, FDG-PET and CT provide accurate findings for evaluating tumor response and also prognosis prediction following first-line nivolumab plus ipilimumab immunotherapy (approximately three cycles).

## INTRODUCTION

Malignant pleural mesothelioma (MPM) is an aggressive neoplasm and affected patients have low survival rates. For over ten years, the primary treatment choice has been platinum-based chemotherapy, though long-term survival remains poor in cases that underwent treatment with cisplatin in combination with pemetrexed [1]. Bevacizumab is listed in the National Comprehensive Cancer Network Clinical Practice Guidelines in Oncology (NCCN guidelines) as a treatment option, though its use as part of combination therapy has not been approved for MPM treatment by the United States Federal Drug Administration (USFDA) or any other international regulatory agency [2]. Although recent developments of immune checkpoint inhibitors (ICIs) has increased interest in immunotherapy use for MPM cases, early results obtained in clinical trials for single immune check-point inhibition treatment have not been conclusive [3].

Immune checkpoint inhibitor antibodies include nivolumab and ipilimumab, which are fully human anti-programmed cell death 1 (PD-1) anti-cytotoxic T-lymphocyte 4 (CTLA-4) antibodies that are distinct, yet also possess action mechanisms that are complementary. Ipilimumab is known to induce T-cell proliferation and de-novo anti-tumor T-cell responses, including in memory T cells, while nivolumab has been shown to restore existing anti-tumor T cell functions [4]. Treatment with both has been approved for melanoma and renal cell carcinoma, and also non-small-cell lung cancer cases. For second-line or later MPM therapy, the NCCN guidelines note that nivolumab, either with or without ipilimumab, is a preferred option for treatment (category 2A). That recommendation was made following results presented from three phase 2 trials [5–7], including the IFCT-1501 MAPS2 trial that featured a multicentre open-label randomised non-comparative design and obtained encouraging results from that combination therapy.

The phase 3 study CheckMate 743 examined first-line nivolumab plus ipilimumab for efficacy and safety in cases with unresectable MPM, and compared the results to platinum plus pemetrexed chemotherapy [8]. Three hundred three patients who received nivolumab and ipilimumab showed improvement in overall survival (OS), as those had a median term of 18.1 months (95% confidence interval (CI) 16.8–21.5) as compared to 302 patients who underwent chemotherapy (14.1 months, 95% CI 12.5–16.2, hazard ratio (HR) 0.74; *p* = 0.002). Furthermore, patients with a non-epithelioid histology showed greater benefit than those with an epithelioid histology. Following presentation of those results, first-line treatment consisting of nivolumab and ipilimumab was approved by the USFDA in October 2020 for adults with unresectable MPM. Additionally, nivolumab plus ipilimumab is recommended in the NCCN guidelines as a preferred first-line option (category 2A) for biphasic or sarcomatoid histology patients, and is also considered as an alternative for epithelioid histology cases.

Systemic treatment response must be adequately assessed for effective cancer treatment management. A crucial factor is determination of responsiveness to systemic therapy by the tumor in order to determine harmful effects and also mortality risk. No known findings obtained with [^18^F]fluorodeoxyglucose positron emission tomography/computed tomography (FDG-PET/CT) or CT used for determining MPM patient response to combined ICI therapy have been presented. The present retrospective study was conducted to examine the effectiveness of FDG-PET criteria, i.e., immunotherapy-modified positron emission tomography response criteria in solid tumors (imPERCIST) [9], with morphological CT criteria, i.e., modified response evaluation criteria in solid tumors (mRECIST) [10], to evaluate patients with unresectable MPM undergoing nivolumab plus ipilimumab combination therapy as first-line treatment regarding response and prognosis prediction.

## RESULTS

### Treatment response assessment

**Figure 1:**
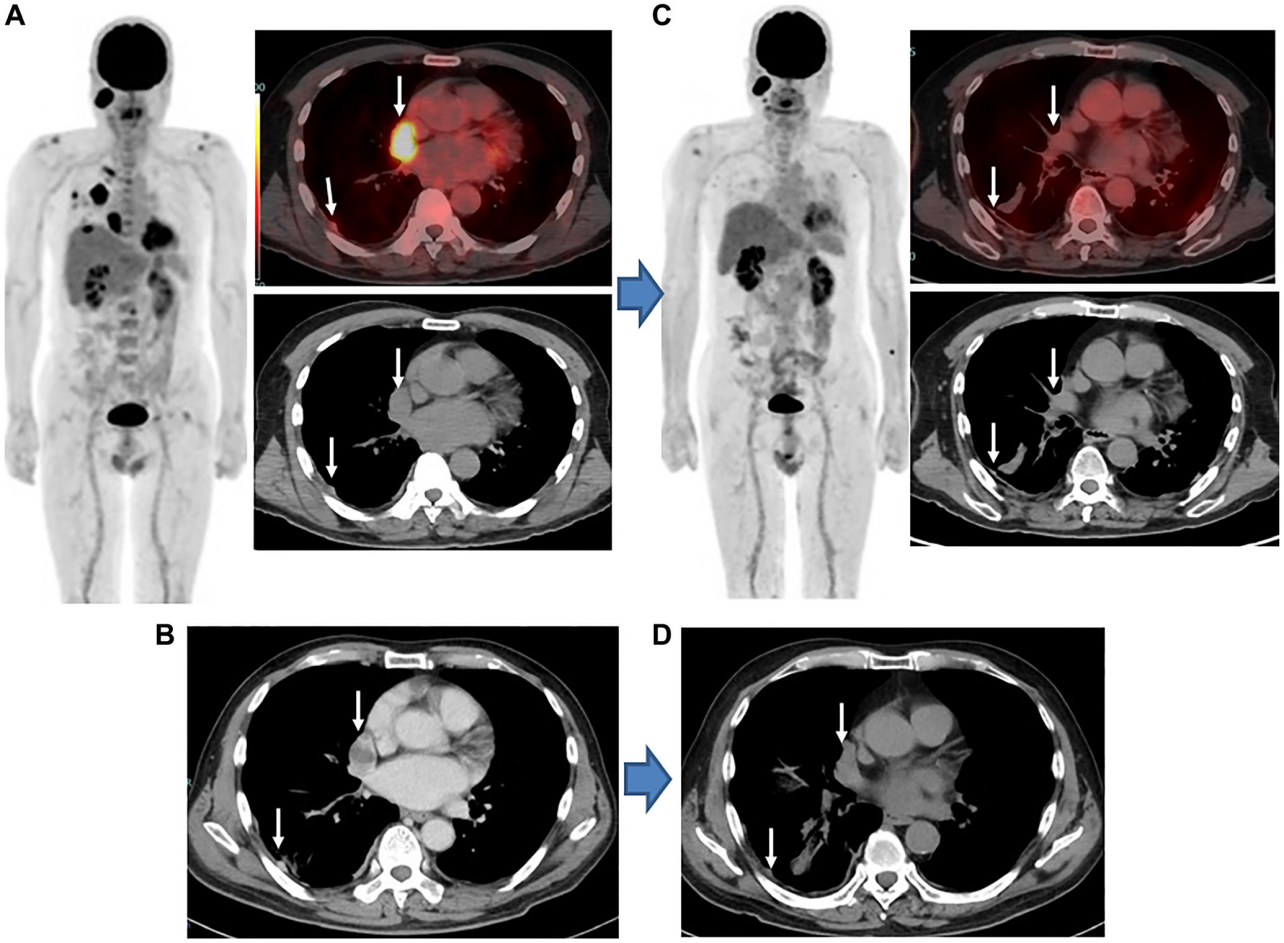
Sarcomatoid MPM, stage IB (cT3N0M0), man, 70 years old. (**A**) Findings obtained with FDG-PET/CT prior to combined nivolumab and ipilimumab immunotherapy indicated several areas in right pleural lesions with high levels of FDG uptake (arrows). Tumor-like FDG uptake in parotid gland on the right side indicates a Warthin tumor. (**B**) Findings obtained with contrast-enhanced CT findings prior to combined nivolumab and ipilimumab immunotherapy indicated a mass-forming thickness in the right pleura (arrows). (**C**) Following four cycles of combined nivolumab and ipilimumab treatment, FDG-PET/CT findings showed FDG uptake disappearance (lower than mean liver activity) in pleural lesions (arrows). (**D**) Following four cycles of combined nivolumab and ipilimumab, non-contrast CT findings showed mild improvement in pleural lesions (arrows). imPERCIST findings showed CMR, while mRECIST indicated PR classification, with a decrease in total size of six pleural lesions perpendicular to the chest wall of 47.1%. At 12.1 months following initiation of combined immunotherapy, progression was noted and the patient died at 16.8 months.

**Figure 2:**
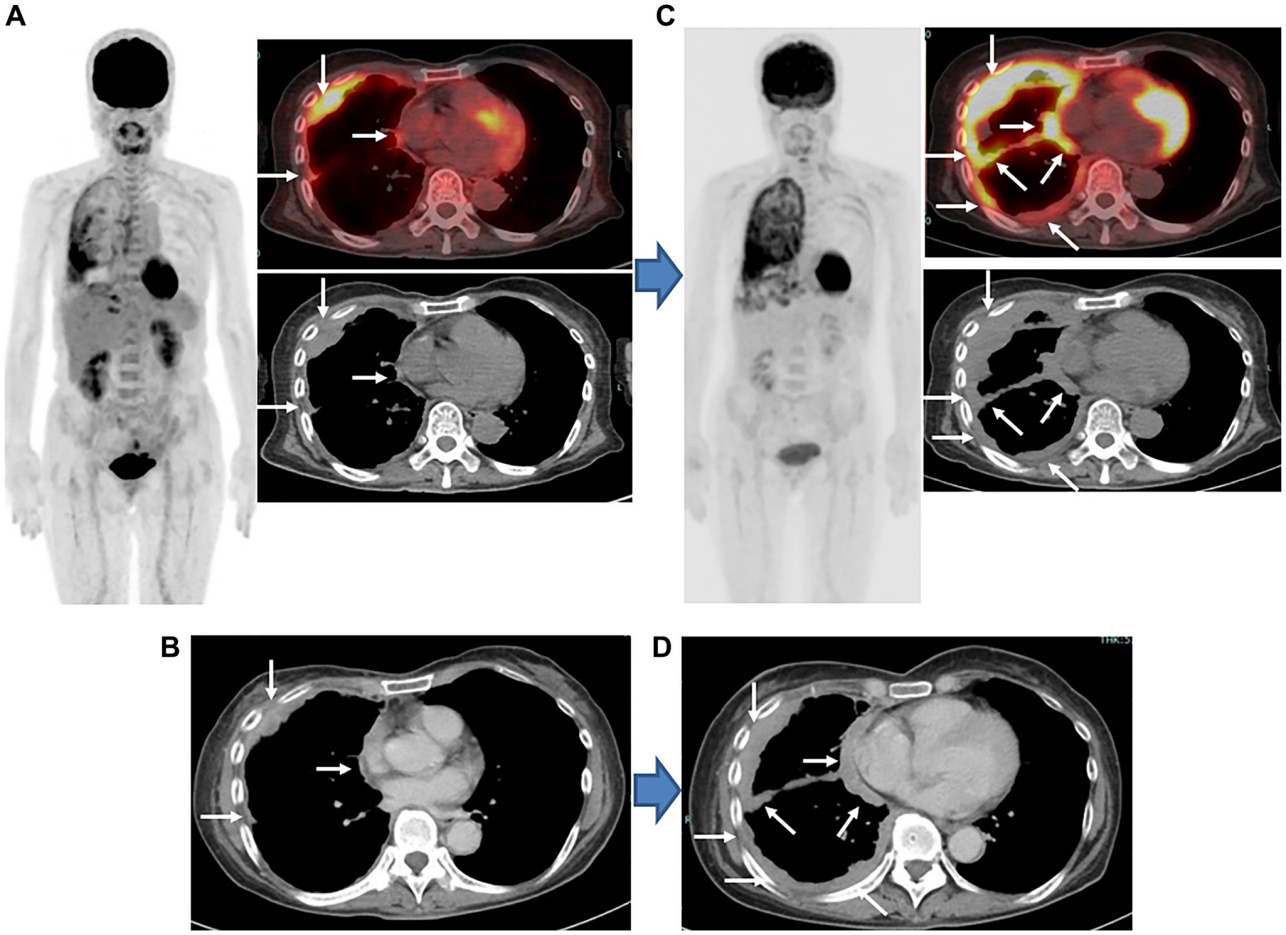
Epithelioid MPM, stage IIIB (cT4N0M0), woman, 64 years old. (**A**) Findings obtained with FDG-PET/CT prior to combined nivolumab and ipilimumab immunotherapy indicated several areas in right pleural lesions with strong FDG uptake (arrows). (**B**) Findings obtained with contrast-enhanced CT findings prior to combined nivolumab and ipilimumab immunotherapy indicated multiple areas of mass-forming thickness in right pleura (arrows). (**C**) Following three cycles of combined nivolumab and ipilimumab treatment, FDG-PET/CT findings showed remarkable progression of multiple pleural lesions and also revealed new pleural lesions (arrows). (**D**) Following three cycles of combined nivolumab and ipilimumab, contrast-enhanced CT findings also showed remarkable progression of pleural lesions and also revealed new lesions (arrows). imPERCIST findings showed PMD, while mRECIST indicated PD, due to remarkable progression and also new lesions. FDG-PET/CT results indicated an increase in SULpeak sum of 33.8% for the five highest level pleural lesions, while CT results showed an increase in the sum size of six pleural lesions perpendicular to the chest of 146.3%. Chemotherapy (cisplatin and pemetrexed) was started, though the patient died after 2.1 months.

Talc pleurodesis was performed prior to the second FDG-PET/CT scan in 11 patients (42.3%), which showed pleural calcification in CT and non-specific FDG uptake in FDG-PET/CT findings, determined to indicate a benign granulomatous inflammatory process and not a recurrent MPM lesion. Treatment efficacy based on imPERCIST using FDG-PETCT findings was progressive metabolic disease (PMD in nine (34.6%), stable metabolic disease (SMD) in eight (30.8%), partial metabolic response (PMR) in eight (15.4%), and complete metabolic response (CMR) in five (19.2%) patients, and that with use of the mRECIST criteria with diagnostic CT findings was progressive disease (PD) in nine (34.6%), stable disease (SD) in nine (34.6%), partial response (PR) in seven (26.9%), and complete response (CR) in one (3.8%). A high level of concordance between the imPERCIST and mRECIST results was noted (κ = 0.827), though a greater percentage of patients (15.4%) were judged correctly using the imPERCIST criteria (Table 1). Results of two representative cases are presented in Figures 1 and 2.

**Table 1:** Comparison of treatment response assessments in imPERCIST and mRECIST in non-epithelioid group

		imPERCICIST (FDG-PET/CT)				Total
		CMR	PMR	SMD	PMD	
mRECIST (CT)	CR	1	0	0	0	1
	PR	2	2	0	0	4
	SD	0	0	3	0	3
	PD	0	0	0	2	2
	Total	3	2	3	2	10

Data are presented as numbers. Abbreviations: imPERCIST: immunotherapy-modified Positron Emission Tomography Response Criteria in Solid Tumors; FDG-PET/CT: [^18^F]fluorodeoxyglucose positron emission tomography/computed tomography; CMR: complete metabolic response; PMR: partial metabolic response; SMD: stable metabolic disease; PMD: progressive metabolic disease; mRECIST: modified Response Evaluation Criteria in Solid Tumors; CR: complete response; PR: partial response; SD: stable disease; PD: progressive disease.

Assessment of treatment response was also performed for the epithelioid (*n* = 16) and non-epithelioid (*n* = 10) histology groups. In the epithelioid group, imPERCIST results showed PMD in seven, SMD in five, PMR in two, and CMR in two patients, while mRECIST results showed PD in seven, SD in six, PR in three, and CR in none (Table 1). Although the concordance between imPERCIST and mRECIST was high (κ = 0.805), the percentage of patients shown to be CMR was greater with imPERCIST(12.5%). In the non-epithelioid group, imPERCIST results showed PMD in two, SMD in three, PMR in two, and CMR in three patients, and mRECIST results showed PD in two, SD in three, PR in four, and CR in one (Table 1). Again, though the level of concordance was high (κ = 0.828), a greater percentage was shown to be CMR by imPERCIST (20.0%).

### PFS

**Figure 3:**
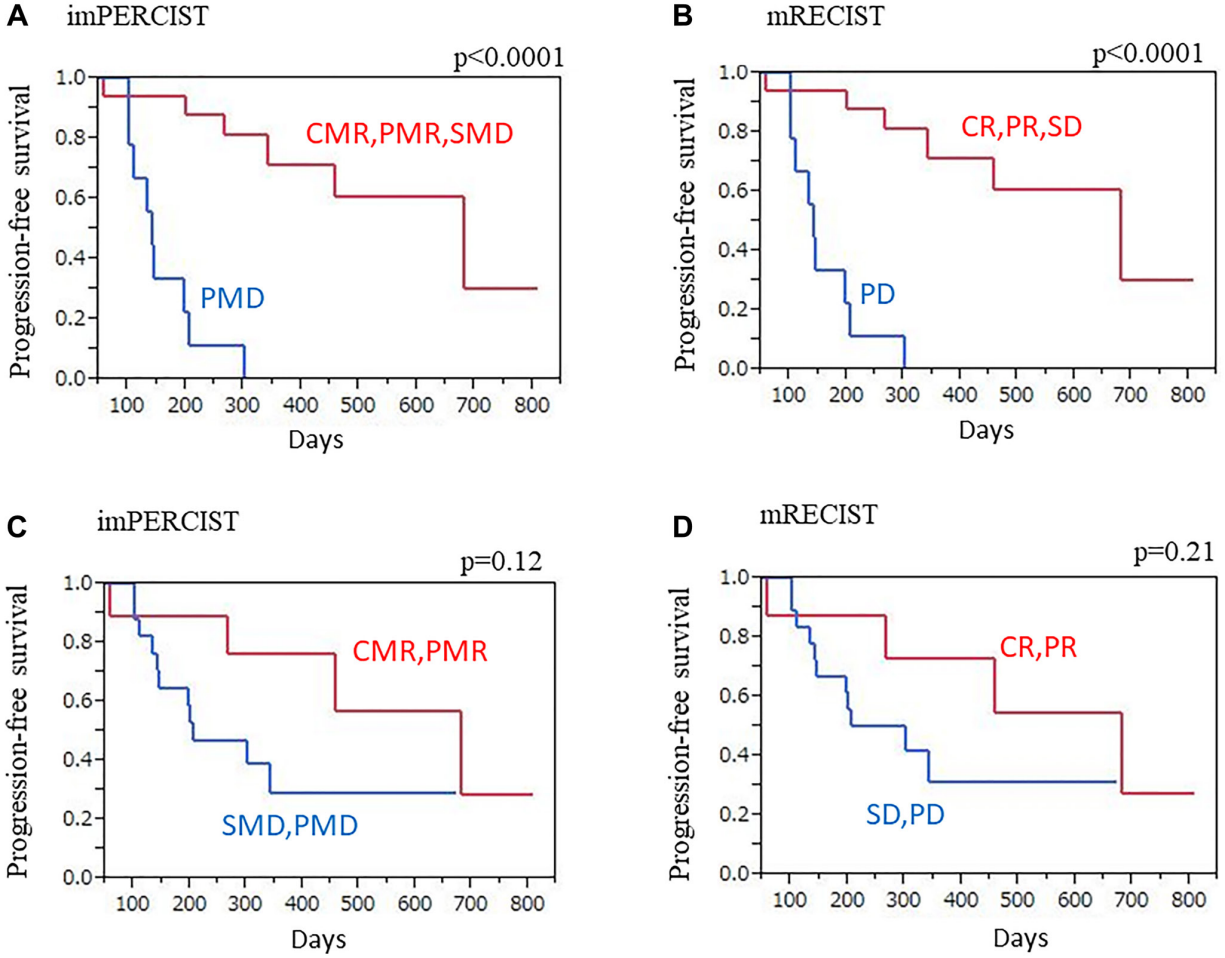
PFS with imPERCIST and mRECIST, Kaplan-Meier curves. (**A**) imPERCIST findings showed that patients without progression (CMR/PMR/SMD) had significantly longer PFS as compared to those with PMD (*p* < 0.0001). (**B**) mRECIST findings showed that patients without progression (CR/PR/SD) had significantly longer PFS as compared to those with PD (*p* < 0.0001). (**C**) imPERCIST findings showed longer PFS in responders (CMR/PMR) as compared to non-responders (SMD/PMD), though not a significant difference (*p* = 0.12). (**D**) mRECIST findings showed longer PFS in responders (CR/PR) as compared to non-responders (SD/PD), though not a significant difference (*p* = 0.21).

A median term of 8.9 months (1.9–26.9 months) for progressive disease development was noted in 15 (57.7%) of the 26 patients by CT or FDG-PET/CT. No brain metastasis was seen in brain MRI findings. Patients without progression (imPERCIST: CMR/PMR/SMD, mRECIST: CR/PR/SD) were found to have significantly longer progression-free survival (PFS) than either PMD or PD patients (imPERCIST: *p* < 0.0001, mRECIST: *p* < 0.0001) (Figure 3A, 3B). Similarly, patients classified as responders (imPERCIST: CMR/PMR, mRECIST: CR/PR) had longer PFS than the non-responders (imPERCIST: mRECIST: SMD/PMD, SD/PD), though it was not a significant difference (imPERCIST *p* = 0.12, mRECIST *p* = 0.21) (Figure 3C, 3D).

### OS

**Figure 4:**
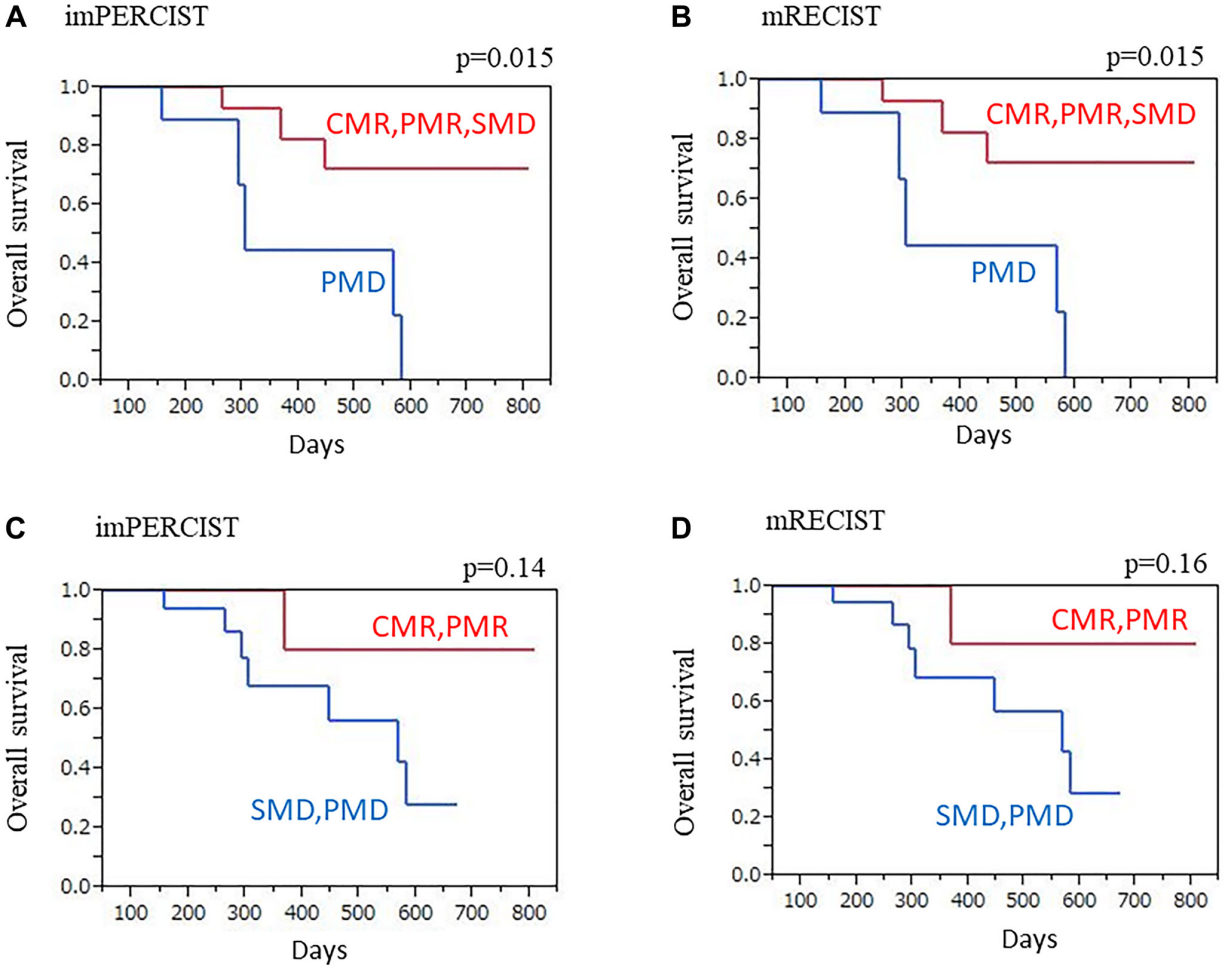
OS with imPERCIST and mRECIST, Kaplan-Meier curves. (**A**) imPERCIST findings showed significantly longer OS in patients with no progression (CMR/PMR/SMD) as compared to those with PMD (*p* = 0.015). (**B**) mRECIST findings showed significantly longer OS in patients with no progression (CR/PR/SD) as compared to those with PD (*p* = 0.015). (**C**) imPERCIST showed longer OS in responders (CMR/PMR) as compared to non-responders (SMD/PMD), though not a significant difference (*p* = 0.14). (**D**) mRECIST showed longer OS in responders (CR/PR) as compared to non-responders (SD/PD), though not a significant difference (*p* = 0.16).

After a median 10.0 months (2.4–27.0 months), eight (30.8%) patients in the entire cohort died from MPM. Based on imPERCIST (CMR/PMR/SMD) and mRECIST (CR/PR/SD), those without progression had significantly longer OS than PMD and PD patients (imPERCIST *p* = 0.015, mRECIST *p* = 0.015) (Figure 4A, 4B). Similarly, longer OS was noted in patients classified as responders (imPERCIST: CMR/PMR, mRECIST: CR/PR/SD) as compared to non-responders (imPERCIST: SMD/PMD, mRECIST: SD/PD), though it was not a significant difference (imPERCIST *p* = 0.14, mRECIST *p* = 0.16) (Figure 4C, 4D).

## DISCUSSION

These findings are the first known to be presented for comparisons of FDG-PET/CT (imPERCIST) and CT (combined mRECIST and RECIST 1.1) criteria as evaluation of tumor response to first-line combined immunotherapy, including nivolumab and ipilimumab, in patients with unresectable MPM and also prognosis prediction. Analyses of the present patient cohort showed that each of the methods provided accurate findings to evaluate tumor response, and also PFS and OS, though the FDG-PET/CT criteria demonstrated a slight superiority. For evaluating the viability of findings of a remaining tumor without unusual FDG uptake during treatment or after, FDG-PET/CT is considered to be an effective tool that provides accurate results for clear diagnosis of CMR. Using the imPERCIST criteria, CMR classification was noted in four (15.4%) of the patients in the present study, which was not indicated by diagnostic CT criteria (mRECIST) results. This tendency was observed in both the epithelioid (12.5%) and non-epithelioid (20.0%) histology groups.

FDG-PET is generally considered as a useful metabolic evaluation tool, while it is also thought to have an emerging role for assessment of systemic therapy response. Metabolic activity changes typically occur sooner than tumor size change during systemic therapy, thus FDG-PET findings can be used for detection of such a response earlier than a measurable change is detected by CT, thus allowing for revealing of response or non-response early during treatment. In addition, FDG-PET/CT findings can be useful for detection of metastasis in bone or muscle, or in tiny lymph nodes, and also small areas of dissemination.

The present results showed significant differences between the criteria for imPERCIST and mRECIST for PFS and OS assessments, which can be used to differentiate between progressive and non-progressive disease. However, the differences between progressive and stable disease, and complete and partial response were not significantly different. Therefore, accurate findings for diagnosis of progressive disease obtained in imaging examinations are clinically important for patient care because of the possibilities of poor prognosis as well as use of alternative therapy.

A previous study investigated 30 patients with recurrent MPM, and compared evaluations of nivolumab treatment response using FDG-PET/CT and diagnostic CT results, as well as prediction of PFS and OS. Results obtained with both were found to be acceptable for accurate evaluation of tumor response following nivolumab administration, and also predicting progression in those patients. It was concluded that FDG-PET/CT findings indicated greater percentages of patients as CMR (16.7%) and PMD (10–13.3%) as compared with CT [11]. In addition, several studies have compared the usefulness of CT and FDG-PET/CT results for evaluating response to treatment in unresectable MPM patients who underwent three chemotherapy cycles, though it remains controversial whether either is superior for that purpose. Veit-Haibach et al. evaluated 41 MPM patients for treatment response following three cycles of pemetrexed and platinum-based chemotherapy [12], and found that CT response had a significant relationship to OS in those cases (*p* = 0.001), while SUVmax response did not (*p* = 0.61). An analysis conducted by Kitajima et al. included 75 MPM patients who underwent cisplatin and pemetrexed treatments [13], and the findings indicated that after three chemotherapy cycles FDG-PET/CT results had greater accuracy as compared to mRECIST results for evaluation of tumor response to chemotherapy, as well as for prediction of prognosis of MPM patients who showed a non-resectable condition.

Limitations of this study include its retrospective design, analysis of results obtained at a single center, and low number of samples, thus making it difficult to generalize the findings and perform analysis without statistical errors. To more clearly clarify the usability of FDG-PET/CT and CT findings for making treatment decisions, and also predicting long-term outcome in a clinical setting, a larger prospective multicenter trial with a greater number of patients will be necessary. Results of such a trial could provide important information to differentiate between stable and progressive disease with use of FDG-PET/CT and CT imaging findings.

In summary, in the present cases, FDG-PET/CT (imPERCIST) as well as CT (mRECIST) results were found useful for evaluating the response of tumors to the combination of nivolumab and ipilimumab (approximately three cycles) used as first-line therapy, and also progression prediction in patients with unresectable MPM. A high level of concordance between imPERCIST and mRECIST was noted (κ = 0.827), though when compared to results obtained with CT, a greater percentage of patients (15.4%) were judged as CMR using results obtained with FDG-PET/CT, indicating its accuracy for tumor viability evaluation.

## MATERIALS AND METHODS

### Patients

A local review board provided approval for conducting this retrospective study (no. 1894). Patient written informed consent was not required by the ethics committee, because of its retrospective observational design. Patients considered eligible for analysis had received a histological diagnosis of MPM, were not considered to be a surgery candidate, and were anticipated to receive first-line combined immunotherapy with nivolumab and ipilimumab. A total of 26 MPM patients (23 males, 3 females; median 73.5 years old, range 63–85 years) who had been referred to our institution for examinations between September 2021 and December 2023 were included. Each underwent diagnostic CT and FDG-PET/CT baseline examinations, and also after receiving approximately three cycles of combined immunotherapy treatment (2 cycles in three, 3 cycles in 17, 4 cycles in six patients). Table 1 shows patient and also tumor characteristics. Talc pleurodesis was performed before the treatment in 11 patients (42.3%), and the time interval between talc pleurodesis and the second FDG-PET/CT scan was 4.6–6.3 months (median 5.5 months). Baseline FDG-PET/CT results were obtained at a median 1.2 months (0.7–2.4 months) and diagnostic CT results at 1.6 months (0.9–2.5 months) prior to starting combined immunotherapy, as well as during that treatment. For each patient, the time interval between FDG-PET/CT and diagnostic CT examinations conducted for baseline findings, and also between those performed during combined immunotherapy was less than two weeks.

As for the immunotherapy regimen, nivolumab was given every two weeks at 3 mg/kg and ipilimumab every six weeks at 1 mg/kg, with administration continued until notification of disease progression or findings indicating toxicity that was not acceptable. Treatment-related adverse events occurred in four (15.4%) (enteritis in two, dermatitis in one, encephalitis in one) of the 26 patients who were enrolled.

CT, FDG-PET/CT, and brain magnetic resonance imaging (MRI) results obtained during the follow-up period were accessed for determining disease recurrence, metastasis, and progression diagnosis. When physical findings indicated progression or recurrence, CT or FDG-PET/CT was used to evaluate the state of the entire body, while brain MRI screening was also conducted. When progression or recurrence was not suspected, imaging examinations performed every six to 12 months were used for surveillance. In cases with discontinuation of combination immunotherapy, alternative chemotherapy (cisplatin/carboplatin and pemetrexed) was subsequently attempted.

### FDG-PET/CT

All FDG-PET/CT examinations were conducted with Ingenuity TF (Philips Medical Systems, Eindhoven, The Netherlands) or Discovery IQ (GE Healthcare, Waukesha, WI, USA) scanners, with the same device used for both baseline and follow-up scans in individual patients. Details regarding the FDG-PET/CT procedures used have been described [11].

### Diagnostic CT

A total of 52 scanning examinations were performed. For 48 of those, pre-contrast and contrast-enhanced CT images of the neck, chest, abdomen, and pelvis were obtained by use of a 128-detector row CT (SOMATOM Definition AS) at 120 kV (effective mA 220 (CAREDose4D), beam pitch 0.6, collimation 1.2 × 32 mm, B31 + medium smooth + image reconstruction). Details regarding the contrast-enhanced CT procedures used have been presented [11]. Non-contrast enhanced CT was used for the other four examinations.

### Image analysis

A nuclear medicine physician with board certification and 14 years of experience performing oncologic FDG-PET/CT examinations reviewed the obtained images in a retrospective manner. To assist attending clinicians performing monitoring of treatment response, GI-PET (AZE Co., Ltd., Tokyo, Japan), a commercially available software package devised for harmonizing standardized uptake values (SUVs) found with various PET/CT systems using phantom data [14], was used. The target lesion maximum concentration (injected dose/body weight) was employed to determine maximum SUV (SUVmax). SUVpeak was calculated based on region of interest (ROI) with a diameter of 1.2 cm selected on the hottest site of the tumor, then normalized to SULpeak (SUVpeak × (lean body mass)/(total body mass)).

CT images used for diagnosis were retrospectively reviewed by a radiologist with 14 years of experience with CT and board certified. Analyses of coronal, axial, and sagittal section images were performed, with winding applied as appropriate.

### imPERCIST

Therapeutic response with use of imPERCIST [9] was calculated with SUL values determined based on an ROI measured at 1.2 cm in diameter on the target lesion. To determine patient condition, CMR, defined as fully resolved FDG uptake within the target lesion (lower than mean liver activity and indistinguishable from blood-pool level in background); PMR, SULpeak reduction of ≥30% and absolute drop of 0.8 SULpeak units in the target lesion (greatest uptake by lesion in each PET/CT scan); PMD, 30% increase in SULpeak of FDG uptake increased by ≥30% and absolute increase of 0.8 SULpeak units, with TLG increase >75%; and SMD, not classified as CMR, PMR, or PMD. The SULpeak sum included new lesions when the uptake level was higher as compared to existing target lesions or when baseline scan results indicated fewer than five target lesions.

### Modified RECIST (mRECIST)

The mRECIST criteria were used for CT image evaluations [10], with tumor thickness determined perpendicular to the chest wall or mediastinum in two positions at three levels. Response Evaluation Criteria in Solid Tumors, version 1.1 (RECIST 1.1), was used for assessment of morphological response of nonplural lesions [15]. Based on modified RECIST and RECIST 1.1 results, a decrease in largest diameter sum ≥30% was considered PR, while a ≥20% increase was considered to indicate PD. A change between PR and PD of <−30% to <+20% was noted as SD. CR was determined for cases with nonplural target lesion disappearance with the shortest axis <1 cm for lymph nodes, while appearance of a new lesion indicated PD. Worst objective response was used as the final classification shown by CT for comparisons of results of mRECIST and RECIST 1.1.

### Statistical analysis

Assessments of concordance between the two methods examined in the present study was were performed based on Cohen’s k coefficient [16], with results showing slight (k < 0.21), fair (k = 0.21–0.40), moderate (k = 0.41–0.60), substantial (k = 0.61 = 0.80), or nearly perfect (k > 0.80) agreement compared. The time period between combined immunotherapy to disease progression (based on radiological and/or clinical examination findings) or death regardless of cause was used for determination of PFS. No evidence of progressive disease at the final follow-up examination resulted in patient censoring. Time from start of immunotherapy until death regardless of cause was used to define OS. Surviving patients were censored on the date of the final follow-up examination, with alive with disease or no evidence of progression used as the classification. Actuarial survival curves were generated with the Kaplan-Meier method, with log-rank test results used to examine differences between groups. All statistical analyses were performed using SAS, version 9.3 (SAS Institute Inc., Cary, NC, USA), with significance indicated by a *p*-value < 0.05.

## Abbreviations

CI: confidence interval
CM: complete response
CMR: complete metabolic response
CTLA-4: anti-cytotoxic T-lymphocyte 4
FDG-PET/CT: [^18^F]fluorodeoxyglucose positron emission tomography/computed tomography
HR: hazard ratio
ICI: immune check-point inhibitor
imPERCIST: immunotherapy-modified PERCIST
iPERCIST: immune PERCIST
MPM: malignant pleural mesothelioma
mRECIST: modified response evaluation criteria in solid tumors
MRI: magnetic resonance imaging
NCCN: National Comprehensive Cancer Network
OS: overall survival
OSEM: ordered-subset expectation maximization
PD: progressive disease
PD-1: anti-programmed cell death 1
PERCIST: positron emission tomography response criteria in solid tumors
PFS: progression-free survival
PMD: progressive metabolic disease
PMR: partial metabolic response
PR: partial response
SD: stable disease
SMD: stable metabolic disease
SUV: standardized uptake values
USFDA: United States Federal Drug Administration
